# Coronary CT angiography and serum biomarkers are potential biomarkers for predicting MACE at three-months and one-year follow-up

**DOI:** 10.1007/s10554-022-02646-4

**Published:** 2022-08-08

**Authors:** Honglei Huang, Fei Ye, Yuanmao Huang, Guiyun Ye, Jiarui Zhu, Xidi Chi, Gaofeng Zhang

**Affiliations:** 1 Department of Radiology, The Affiliated Nanping First Hospital of Fujian Medical University, Nanping, China; 2 Department of Laboratory, The Affiliated Nanping First Hospital of Fujian Medical University, Nanping, China; 3 Department of Radiology, Affiliated Hospital of Zunyi Medical University, Zunyi City, China

**Keywords:** Coronary artery disease, Coronary CT angiography (CTA), Serum proteins, Potential biomarkers, Major adverse cardiac events (MACE)

## Abstract

**Aims:**

To assess the prognostic value of coronary computed tomography angiography (CTA) and serum biomarkers for the prediction of major adverse cardiac events (MACE) at three-month and one-year follow-ups.

**Methods and Results:**

A total of 720 patients with acute chest pain and normal electrocardiography (ECG) were included in the prospective cohort study. These patients received both coronary CTA screening and serum biomarkers testing, followed by three-month and one-year follow-ups for the occurrence of major adverse cardiac events (MACE). The primary outcome was the occurrence of MACE, which is defined as acute coronary syndrome (ACS), nonfatal MI, and all-cause mortality. The MACE rate was 17.8% (128 cases) and 25.2% (182 cases) at three-months and one-year follow-up. ApoB/apoA1(OR = 7.45, P < 0.001) and the number of atherosclerotic vessels (OR = 2.86, P < 0.001) were independent predictors for MACE at the three-month follow-up, so were apoB/apoA1 (OR = 5.23, P = 0.003), Serum amyloid protein A (SAA, OR = 1.04, P < 0.001) and the number of atherosclerotic vessels (OR = 2.54, P < 0.001) at the one-year follow-up. While apoB/apoA1 suggested its sensitivities of 84% for predicting MACE at three-month follow-ups, the number of atherosclerotic vessels had 81% specificity at one-year follow-up.

**Conclusions:**

Among patients with acute chest pain and normal ECG, apoB/apoA1, SAA and the number of atherosclerotic vessels are the most powerful predictors of MACE at three-month and one-year follow-ups.

## Introduction

Coronary artery disease (CAD), often coming with angina pectoris, myocardial infarction, and ischemic heart failure is one of the leading causes of mortality and socioeconomic burden [1]. Major adverse cardiac event (MACE) is used to define a composite cardiovascular outcome, thus in part reflecting the degree of CAD [2].

Coronary computed tomography angiography (CTA) adopts a valuable non-invasive approach for early diagnosis of CAD, such as excluding major CAD [3]. Coronary artery stenosis can be evaluated by volume mapping, multiplane reconstruction and cross-sectional images, achieving an accuracy of 89% for stenosis detection with sensitivity, specificity and the positive predictive value of 97%, 72% and 88%, respectively [4]. Over the past 20 years, many researchers have tried to develop the prognostic value of CTA parameters, including increasing the accuracy itself, stratifying related coronary risks, and predicting cardiovascular outcomes [5, 6]; it has been recognized that degree of coronary stenosis, coronary plaque types and characteristics are valuable prognostic parameters [7].

On the other hand, dyslipidemia is also a significant risk factor of CAD and an effective predictor of MACE. The ratio of low-density lipoprotein (LDL) to high density lipoprotein (HDL), atherogenic index of plasma (AIP), atherosclerosis (AI), triacylglycerol (TG) concentrations and total cholesterol (TC) are reported to quantitatively impact on acute myocardial infarction (MI) that impair long-term clinical outcome [8]. Serum proteins, however, are suggested to have additional diagnostic value for CAD risk stratification. For example, Serum amyloid A (SAA), as the main component of the acute-phase inflammatory response, has been shown to be more sensitive to cardiovascular and non-cardiovascular events than CRP, and patients with elevated SAA levels have a higher incidence of adverse events (mortality, recurrent angina, acute myocardial infarction, stroke, and other vascular events) [9, 10]. Furthermore, the ratio of apolipoprotein B to apolipoprotein A1 (apoB/apoA1) was discovered to be a better predictor for acute MI than TC/HDLc ratio from a nested case-control study [11]. The results of another prospective study showed that apoB/apoA1 ratio was superior to any cholesterol ratios: CHOL/HDL, LDL/HDL or non-HDL/HDL [12].

Although some studies have shown the associations between CTA parameters and MACE as well as between serum proteins and MACE respectively [13, 14], the comparison of CTA parameters to serum proteins with regard to predicting MACE and the independent risk factors of MACE remain unclear. Therefore, the present study aimed to investigate the prognostic values of CTA parameters and serum proteins for the prediction of MACE at three-month and one-year follow-ups among patients with acute chest pain.

## Methods

### Ethics Statement

This study was approved by the Ethics Committee of Fujian Medical University Affiliated Nanping First Hospital. Written informed consent was obtained from all participants. Related study protocol has been registered in Chinese Clinical Trial Registry Center (https://www.chictr.org.cn/enIndex.aspx; registration number: ChiCTR2000034551).

### Study design and population

We prospectively enrolled consecutive participants, who were admitted to the Department of Radiology in Fujian Medical University Affiliated Nanping First Hospital from June 2018 to December 2020. This prospective research was approved by the ethics committee.

Inclusion criteria were patients with: [1] acute chest pain and normal ECG (unknown CAD or high risk of CAD), [2] first medical contact with the onset time less than 3 h, [3] good condition and capability of accepting CTA examination. Patients with a history of percutaneous coronary intervention (PCI), coronary artery bypass grafting (CABG), or other heart valve surgeries were excluded.

Included patients, with self-report of chest pain symptoms including right-sided chest pain, central chest pain, left-sided chest pain, chest tightness, and chest heaviness, mainly came from the Emergency Department (ED). CAD was diagnosed generally according to related clinical symptoms (such as shortness of breath or hypotension) and conventional coronary risk factors (e.g. abetarterial hypertension, dyslipidemia, family history, smoking status, diabetes).

### Baseline evaluation

On enrollment, baseline assessment data included basic information on age, gender, cardiovascular diseases risk factors, symptoms, physical examination with blood pressure. Fasting serum protein was tested in the next morning after the patients presented with chest pain and admitted. BP was measured on the right arm after a rest ≥ 5 min, using an automatic manometer (Omron automatic medical blood pressure monitor HBP-9030) with an appropriate cuff size. Hypertension was defined as a documented history of high blood pressure or treatment with anti-hypertensive medications. Diabetes mellitus was defined by diagnosis of diabetes made previously by a physician and/or use of insulin or oral hypoglycemic agents. Dyslipidemia was defined as known but untreated dyslipidemia, or current treatment with lipid-lowering medications. A positive smoking history was defined as current smoking or cessation of smoking within 3 months of testing.

### Coronary CTA image acquisition and parameters

All patients had normal sinus rhythm and were able to perform the breath-holding required by coronary CTA. Patients were given intravenous Metoprolol to a total dose of 25 mg. If the patient’s heart rate did not fall below 70 bpm, we chose the lowest number as the measured heart rate for coronary CTA. All scans were performed by a 128-slice multi-detector CT scanner (SIMENS, SOMATOM Definition Flash), combined with post-processing workstation (Syngo.via) and probe technology. A contrast test imaging was performed 2 mm from the start of the left main coronary artery to determine the exact time of injection. During the acquisition of coronary CTA, Iodpirol 350 contrast agent 1.1 kg/ml followed by 40ml normal saline was injected. Triphasic contrast-enhanced coronary CTA of the chest was performed, with initiation of the scan immediately superior to the heart and scan termination immediately inferior to the heart. Contrast timing was performed to optimize uniform contrast enhancement of the coronary arteries. The scan parameters were: 128*0.6 mm collimation, tube voltage 100 mV, effective 300 mA. In all cases, auto-modulated mA and ECG dose modulation radiation dose reduction algorithms were employed.

Two senior radiographers recorded atherosclerotic vessels, degree of stenosis and plaque types. In case of disagreement, consensus was reached through consultation. Image data were evaluated by multiple methods including viewing axial source images, multiplanar reformatting, and maximum intensity projection:

1. Coronary stenosis severity was measured according to the Gensini score method, and the stenosis severity was classified as no stenosis (with a score of 0%; recorded as 0), minimal (with a score less than 10%; recorded as 1), mild ≤ 49% (with a scores less than 50%; recorded as 2), intermediate (with a score between 51% and 74%; recorded as 3), or severe (with a score equal to or larger than75%; recorded as 4) per coronary segment (Austen 1975). If one vessel has more than two stenoses, the most serious lesion was taken as the stenosis score of the vessel; if multiple vessels have stenosis, the stenosis scores of each vessel were accumulated.
2. Plaque types were characterized as no plaque (0), calcified [1], mixed [2], non-calcified [3]. Only plaques located at proximal segments were included for the assessment. While calcified plaque was defined as hyper-attenuating lesions, non-calcified plaque was defined as hypo-attenuating lesions [15].
3. Number of atherosclerotic vessels were classified as negative (0), one branch [1], two branches [2] or three branches [3]. The left main artery lesion was calculated when the left anterior descending branch and left circumflex branch appeared at the same time.

### Measurement of serum proteins

After overnight fasting, early morning 3ml venous blood samples were collected on an empty stomach and centrifuged in a biochemical coagulation promoting tube with a centrifugation radius of 14.5 cm. The serum was separated by centrifugation at 3000 R/min for 5 min. The Beckman au5821 biochemical analyzer was used to detect TG and TC. The latex enhanced turbidimetric method was used to detect SAA, lipoprotein (a) [Lp(a)] and high-sensitivity C-reactive protein (hs-CRP). Blood samples for analysis were frozen at -70 °C. Immunoturbidimetry was used to detect HDL-C, LDL-C, ApoB and ApoA1. All operations were carried out in strict accordance with the instructions of the kits.

### Study endpoint and follow-up Procedure

The primary outcome was the occurrence of MACE, which is defined as acute coronary syndrome (ACS), nonfatal MI, unstable angina (UA), target vessel revascularization (TVR) and all-cause mortality. If a patient was recognized as MACE at three-month follow-up, he or she will be automatedly included in the MACE group at one-year follow-up.

The patients having received a quantitative angiographic assessment and serum protein detection would be followed up after three-months and one-year through phone interview or the WeChat app. Each of the participants associated with their family members was informed in advance to seek medical service in our hospital or correspond with us in case of acute chest pain or relative symptoms. And if someone doesn’t visit our hospital or contact us during the follow-up period, we gave him/her a phone call to complete the interview. Incomplete follow-up will be handled with the following algorithm: participants with ongoing visits in the EMR are considered to have complete information and classified based on the data available in the medical record; participants with no ongoing visits are considered lost to follow-up at the point of the last contact. In the event of a discrepancy between a participant’s self-reported event and the medical record, the medical record is considered correct.

### Statistical analysis

All binary relationships were counted by nonparametric Spearman correlation analysis and Wilcoxon rank-sum tests. The P-value of the trend is obtained by the mantel-Haenszel statistic of the frequency and the Jonckheere-Terpstra statistic of the continuous variable. Linear regression was used to analyze the continuous results of multivariable models, such as the number of coronary lesions; Logistic regression analysis of binary results, such as the occurrence of MACE. A stepwise modeling approach is used for all multi variables to develop the best prediction model for the number of coronary lesions or MACE, including known risk factors and other baseline variables available.

Categorized variables were expressed as frequencies and continuous variables as mean ± 1 standard deviation. Classified variables were compared using V2 statistics, and continuous variables were compared using unpaired t test. We designed a risk-adjusted model that included baseline adjustments for cardiac risk factors and stepwise multivariate models at different sites. The relative risk ratio was figured up using a 95% confidence interval (CIs) on account of binomial distribution. Double-tailed *P* < 0.05 was considered statistically significant.

Receiver operating characteristic (ROC) curve was calculated to evaluate the accuracy of coronary CTA parameters and serum proteins in predicting MACE diagnosis. The optimal cut-off value is the detection result closest to the upper left corner of the curve with the highest sensitivity and specificity. The area under the curve directly reflects the diagnostic accuracy of the test. The areas under the ROC curve were compared using the Z-statistic (double-tailed test). *P* value less than 0.05 is considered significant.

## Results

### Baseline characteristics

**Fig. 1:**
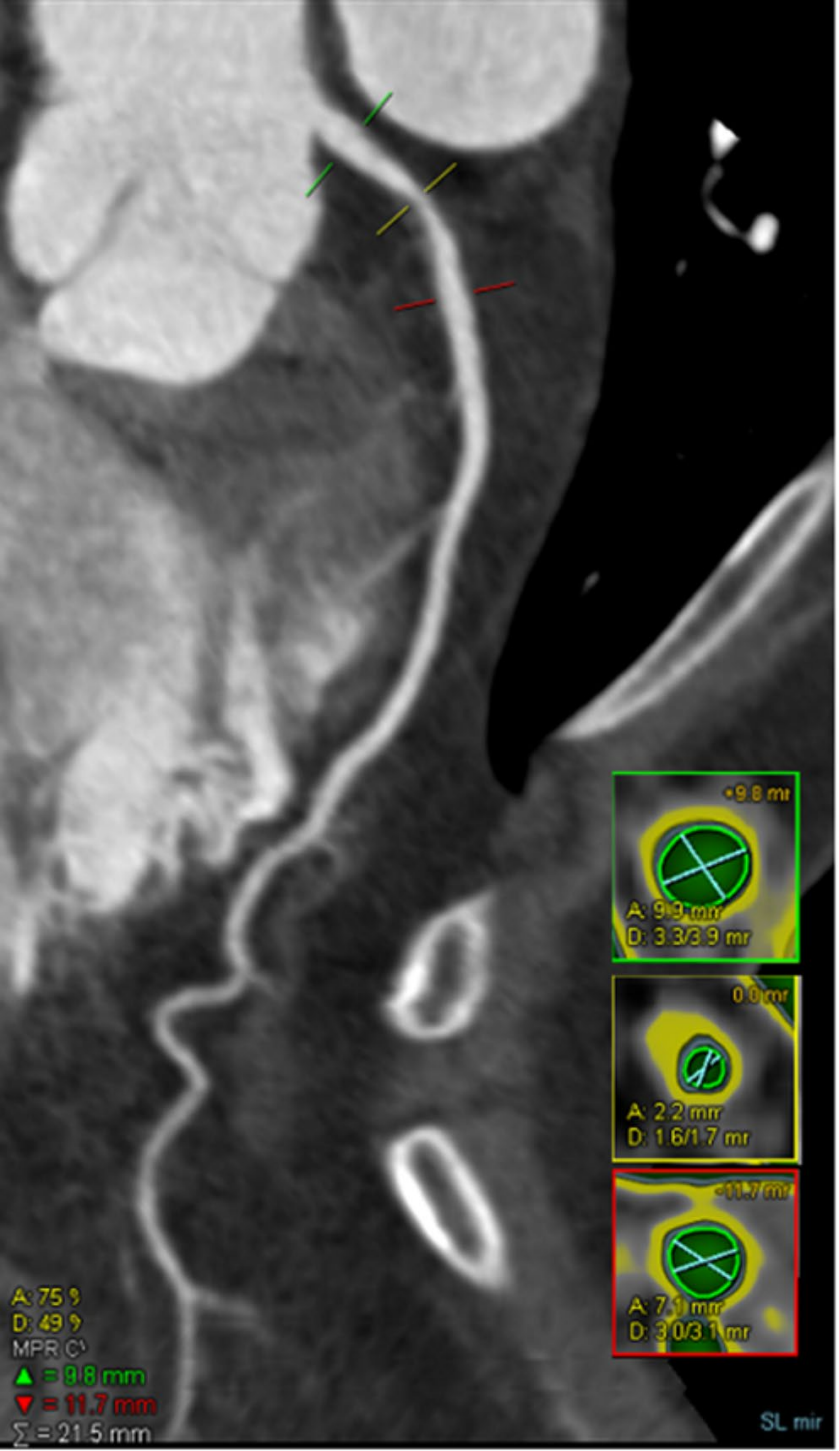
Fifty-eight-year-old male with chest pain for 4 weeks. Non-calcified plaque component and severe stenosis are observed in proximal circumflex artery by CTA. Cross-sectional images show high-risk plaque and stenosis features

720 patients were finally enrolled. The patients’ average age was 62 years old, and almost half of them were men. The results of serum protein tests and coronary CTA parameters were listed in Table 1. There were 128 (17.8%) and 182 (25.2%) MACE at three-months and one-year follow-ups, respectively. A representative case was shown in Fig. 1.

**Table 1:** Demographic Characteristics (n = 720)

Characteristic	Mean, SD, range
Age (year)	62 ± 12 (range, 16–94)
Sex (male, %)	356 (49.4%)
BMI (kg/m2)	24.83 ± 4.43 (range, 15.8–49.9)
Diabetes	216 (30.0%)
Dyslipidemia	467 (64.7%)
Hypertension	325 (45.1%)
Smoking	418 (58.1%)
TC (mmol/L)	4.8 ± 1.2 (1.9–9.7)
TG (mmol/L)	1.7 ± 1.2 (0.5–11.4)
LDL-C (mmol/L)	2.9 ± 1.0 (0.6–7.3)
HDL-C(mmol/L)	1.2 ± 0.3 (0.3–2.1)
ApoB/ApoA1	0.9 ± 0.3 (0.2–1.5)
Lp(a) (mg/L)	205.7 ± 268.3 (3.5-1902.3)
Hs-CRP (mg/L)	4.6 ± 11.2 (0.2-195.4)
SAA (mg/L)	19.7 ± 15.1 (1.0–89.0)
Plaque type	1.6 ± 1.4 (0–3)
Coronary stenosis severity	1.8 ± 1.7 (0–4)
No. of diseased vessels	1.6 ± 1.4 (0–3)

Abbreviations: Apo, apolipoprotein. BMI, body mass index. HDL-C, high density lipoprotein cholesterol. Hs-CRP, high-sensitivity C-reactive protein. LDL-C, low density lipoprotein cholesterol. Lp(a), Lipoprotein(a). SAA, serum amyloid protein A. SD, standard deviation. TC, total cholesterol. TG, total triglyceride

### Univariable logistic regression analysis

In univariable analysis accounting for age, gender, and cardiac risk factors, MACE rates follow up for three months increased with ApoB/apoA1 (OR = 52.17, P < 0.001), SAA (OR = 1.06, P < 0.001), plaque type (OR = 1.81, P < 0.001), stenosis severity (OR = 2.80, P < 0.001) and number of atherosclerotic vessels (OR = 3.69, P < 0.001). At the one-year follow-up, MACE rates increased with age (OR = 1.04, P < 0.001), ApoB/A1 (OR = 50.2, P < 0.001), SAA (OR = 1.00, P < 0.001), plaque type (OR = 2.09, P < 0.001), stenosis severity (OR = 3.26, P < 0.001) and number of atherosclerotic vessels (OR = 3.80, P < 0.001) (Table 2).

**Table 2:** Univariable logistic regression model

Variable	Follow up for three-months			Follow up for one-year		
	OR	95%CI	P	OR	95%CI	P
Age	1.01	0.98–1.03	0.658	1.039	1.023–1.054	0.001
Sex(male)	1.196	0.816–1.754	0.359	1.03	0.736–1.442	0.862
TG	0.813	0.666–0.993	0.043	0.917	0.790–1.066	0.261
TC	0.956	0.816–1.121	0.579	0.962	0.837–1.106	0.586
HDL	0.69	0.332–1.434	0.32	0.689	0.362–1.310	0.256
LDL	1.005	0.823–1.226	0.963	0.976	0.819–1.164	0.79
ApoB/ApoA1	52.304	20.399-134.108	0.0001	50.239	21.715–116.230	0.0001
Lp(a)	1.001	1.000-1.001	0.045	1.001	1.000-1.001	0.007
Hs-CRP	1.008	0.994–1.022	0.28	1.014	0.999–1.029	0.059
SAA	1.058	1.045–1.072	0.0001	1.008	1.071–1.105	0.0001
Plaque type	1.806	1.502–2.171	0.001	2.087	1.763–2.470	0.001
Coronary stenosis severity	2.800	2.274–3.448	0.001	3.258	2.650–4.006	0.001
No. of diseases vessels	3.685	2.784–4.879	0.001	3.803	2.999–4.823	0.001

Abbreviations: Apo, apolipoprotein. HDL-C, high density lipoprotein cholesterol. Hs-CRP, high-sensitivity C-reactive protein. LDL-C, low density lipoprotein cholesterol. Lp(a), Lipoprotein(a). SAA, serum amyloid protein A. OR, odd ratio. SD, standard deviation. TC, total cholesterol. TG, total triglyceride

### Multivariable logistic regression analysis

Multivariable logistic regression analysis was further performed. A total of 3 variables were statistically significant: apoB/apoA1(OR = 7.45, P < 0.001), number of atherosclerotic vessels (OR = 2.86, P < 0.001) at the three-months follow-up; apoB/apoA1 (OR = 5.23, P = 0.003), SAA (OR = 1.04, P < 0.001), number of atherosclerotic vessels (OR = 2.54, P < 0.001) at the one-year follow-up. (Table 3).

**Table 3:** Multivariable logistic regression model

Variable	Follow up for three-months			Follow up for one-year		
	OR	95%CI	P-value	OR	95%CI	P-value
Age	1.000	0.976–1.023	0.969	0.993	0.971–1.015	0.507
ApoB/ApoA1	7.445	2.253–24.602	0.001	5.228	1.789–15.280	0.003
Lp (a)	1.000	1.000-1.001	0.341	1.001	1.000-1.001	0.094
SAA	1.016	0.996–1.035	0.114	1.040	1.019–1.061	0.001
Plaque type	0.816	0.611–1.090	0.168	1.012	0.777–1.318	0.929
Coronary stenosis severity	1.066	0.714–1.590	0.755	1.017	0.700-1.477	0.929
No. of diseases vessels	2.857	1.892–4.315	0.001	2.540	1.768–3.647	0.001

Abbreviations: Apo, apolipoprotein. Lp(a), Lipoprotein(a). SAA, serum amyloid protein A. OR, odd ratio

### Diagnostic accuracy of coronary CTA and serum proteins

**Fig. 2:**
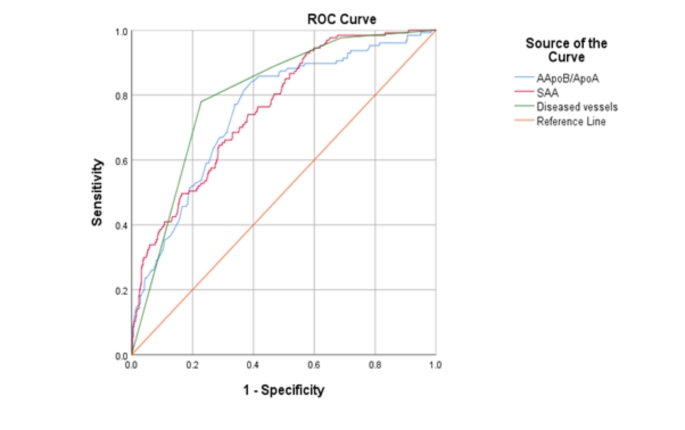
ROC Curve of prediction for three-months MACE

To assess the predictive value of coronary CTA parameters and serum protein levels for prediction of MACE, we conducted a ROC curve analysis to evaluate the diagnostic accuracy of apoB/apoA1, SAA and Number of atherosclerotic vessels (Fig. 2). While apoB/apoA1 presented the highest sensitivities of 84% and 76% at three-months and one-year follow-ups respectively, number of atherosclerotic vessels showed 77% and 81% of the highest values of specificity (Table 4).

**Table 4:** Area Under the Curve

Test Result	Three-months follow-up		One-year follow-up	
Variable(s)	Sensitivity	Specificity	Sensitivity	Specificity
ApoB/ApoA1	84%	60%	76%	65%
SAA	65%	71%	69%	77%
No. of diseased vessels	78%	77%	72%	81%

Abbreviations: Apo, apolipoprotein. Lp(a), Lipoprotein(a). SAA, serum amyloid protein A

**Fig. 3:**
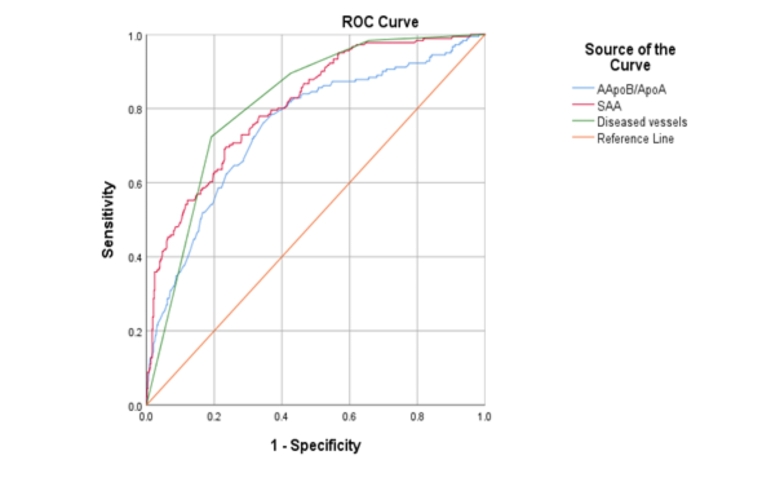
ROC Curve of prediction for one-year MACE

## Discussion

In this study concerning patients with acute chest pain, we evaluated the prognostic value of coronary CTA and serum proteins for MACE at three-months and one-year follow-ups. Despite that previous studies have explored the association between serum proteins (or coronary CTA) and MACE respectively, the comparison of serum proteins to coronary CTA for predicting MACE is lacking. Our results suggest that apoB/apoA1, SAA and number of atherosclerotic vessels are the most powerful MACE predictors and that serum proteins have similar predictive power with coronary CTA, however the latter is more expensive and time-costing.

In recent years, cardiovascular risk assessment has provided standardized guidelines for personalized treatment, which has led to further research on biomarkers. From the perspective of long-term prognosis, it is urgently required to establish new biomarkers that are sufficiently predictive to improve the diagnostic and risk stratification process [16]. Previous studies demonstrate that serum proteins are associated with MACE and atherosclerosis risk factors in patients with acute chest pain [17–19]. Our findings are in line with some of them. A cohort study shows that apoB/apoA1 ratio is an independent predictor for patients with atherosclerotic cardiovascular disease (ASCVD) who are attacked by plague rupture, erosion or thrombus [20]. Accumulating data also suggest that apoB/apoA1 ratio is a better predictor of ACS than LDL-C, HDL-C and TC, which are traditional predictors of CAD and that apoB/apoA1 is strongly associated with ST-segment elevation myocardial infarction (STEMI). However, baseline serum apos is not effective to predict MACE in statin-treated patients during long-term follow-up [21–23]. On the other hand, SAA showed the predictive power of MACE at one-year follow-up (OR = 1.04, P < 0.001). It was reported that high SAA levels are associated with atherosclerotic plaque rupture and may be associated with persistent inflammation within the fibrous cap of the plaque. These pathophysiological findings may be related to the epidemiological observation that circulating inflammatory markers and myeloperoxidase levels predict the risk of future cardiovascular events. Thus, following clinical trials have shown that anti-inflammatory drugs such as aspirin and statins can reduce inflammatory markers and cardiovascular risk [24].

As to coronary CTA parameters, a cohort study assessed the combined MACE endpoints, recognizing an increased risk of MACE when both severe plaque stenosis and mixed plaque components were presented [25]. In our study, nonetheless, plaque types and stenosis severity were not significantly associated with the future occurrence of MACE but only inferred potential risk factors from the univariable analysis. Instead, number of atherosclerotic vessels was associated with the occurrence of MACE both at three-months and one-year follow-ups (OR = 2.86, P < 0.001; OR = 2.54, P < 0.001). Although patients with obvious non-calcifying plaques and severe stenosis are inclined to accept ICA and following treatments of PCI or CABG, those having calcified plagues or mild stenosis possibly receive the undifferentiated treatment compared with high-risk patients, which explains that there is a slight clue but no significant difference of the result. Besides, age seems no association with MACE neither at three-months or one-year follow-up, which is opposite to what is considered commonsense. Although elderly patients have higher risk of the occurrence of MACE, it may be offset by the fact that they are less likely to accept PCI or CABG intervention compared to the younger. Therefore, we can also infer that the presentations of serum proteins and coronary CTA to some extent activate TVR among patients with acute chest pain as a result of significant differences in the short duration of follow-up.

Admittedly, there are some limitations on the study. The impact of coronary CT and serum proteins upon the occurrence of MACE was observed in patients with regardless of previous cardiovascular status and treatment history. For example, patients with complete revascularization are less possible to encounter MACE [26]. Also, the accumulative parameters evaluating plague type and stenosis severity may oversimplify the problem. Innovative coronary risk stratification parameters, such as napkin ring, low-attenuation plaque (LAP), spotty calcification (SC), remodeling index, classification of stenosis, are required to be comprehensively considered in the risk prediction for MACE. Finally, although some results reached statistical significance, the sample size is fairly small. Further studies with larger population and longer follow-up are warranted to verify and enhance the result.

In conclusion, among patients with acute chest pain and normal ECG, apoB/apoA1, SAA and number of atherosclerotic vessels are the most powerful predictors of MACE at three-months and one-year follow-ups, suggesting their integration into cardiovascular risk evaluation and individual prevention.

**Declaration of conflicting interest**.

The authors declare that there is no conflict of interest.
